# Particle size composition characteristics of weathered debris from grey–green slate under the action of freeze–thaw and dry–wet cycles

**DOI:** 10.1038/s41598-023-27888-7

**Published:** 2023-01-25

**Authors:** Jie Wang, Wangcheng Li, Min Mu, Jihong Chen, Yangyang Li, Huaru Liu, Qikun Su

**Affiliations:** 1 Country School of Civil and Hydraulic Engineering, Ningxia University, Yinchuan, 750021 China; 2 Engineering Research Center of Efficient Utilization of Modern Agricultural Water Resources in Arid Regions, Ministry of Education, Yinchuan, 750021 China; 3 State Key Laboratory of Land Degradation and Ecological Restoration in Northwest China, Yinchuan, 750021 China

**Keywords:** Hydrogeology, Environmental impact

## Abstract

The material basis for soil formation is rock weathering debris. Understanding the particle size composition characteristics of rock weathering debris and its impacts is important for improving the soil structure of dry farmland in the central dry zone of Ningxia,China. In this study, the particle sizes of weathered debris collected from grey–green slate after indoor simulations of freeze–thaw and dry–wet cycles tests were examined. The results were as follows: (1) Under 16 treatments, the weathering debris of grey–green slate contained about 10% or less very fine sand and coarse silt, while clay, fine silt, and fine sand were the most abundant sizes (at least 60% of the total). (2) Under each treatment, the average particle size of the grey–green slate weathered debris was 5.52*Ф* (silt grade). The overall skewness was high, but the symmetry was poor. The particle size frequency distribution curve had many broad peaks or multiple peaks. (3) The fractal dimension was associated with very fine silt but had a strong negative correlation with fine sand and medium-coarse sand. The results indicate that fractal dimension can reflect the grain size characteristics of weathered debris. The mineral element content of the grey–green slate somewhat affected the fractal dimension, and it positively correlated with environmental electrical conductivity (EC) and element-leaching amount; it negatively correlated with particle size, temperature, and pH. According to the findings, the fractal dimension can accurately represent the particle size distribution of weathered debris. The generation of grey–green slate weathering debris should be considered in the formation and development of local soil.

## Introduction

The “rock–soil–plant” system is organic. The debris formed during rock weathering has a significant impact on the structural composition and fertility of the soil. The dry farmland in the central arid zone of Ningxia is a typical manifestation of “rock–soil–plant” coexistence. The particle size composition of weathered grey–green slate debris overlying the farmland surface is critical in understanding local dry farmland soil formation and ecological land restoration. According to research, parameters such as particle size composition, mean particle size, median particle size, skewness, standard deviation, kurtosis, and fractal dimension are commonly used to reflect specific physical properties of soils^1,2^. To some extent, a higher clay content in the soil correlates to more agglomerates and an improvement in the soil water retention capacity^3^. The coarser the soil particles, the lower the organic carbon content of the soil and the relative instability of its physicochemical properties and structure^4^. The standard deviation can reflect the degree of soil particle sorting, and the fractal dimension can be used to characterize soil structure and properties^5^. Weathered debris is the starting point for soil formation, and the particle size composition somewhat influences soil structure.

Most scholars^6–9^ have investigated the particle size composition of weathered debris by analysing the particle size characteristics of sediments. These investigators based their findings on the particle size characteristics of deposits to reflect the depositional environment in which they formed. Wind and sand are primarily responsible for differences in particle size characteristics of sediments in the Yarlung Tsangpo River, where the depositional environment is generally fluvial and lacustrine^10^. According to an analysis of its sediment grain size parameters, the Dunhuang Yardang landform is the result of an accumulation of multiple sediments formed under different depositional environments and dynamical regimes. i.e., wind, fluvial, and lacustrine environments^11^. Few studies have been published on the particle size characteristics of weathered debris in relation to soil evolution. Thus, it is critical to clarify the particle size characteristics of weathered grey–green slate waste in central Ningxia to determine the causes of ecological fragility and soil erosion severity. The most common local grey–green slate was chosen as the subject of this study. The weathering debris produced by the grey–green slate following the freeze–thaw and dry–wet cycles was collected; then, the particle sizes were analyzed to provide a scientific foundation for ecological restoration and sustainable development in Ningxia's central dry zone.

## Material and methods

### Study area

**Figure 1:**
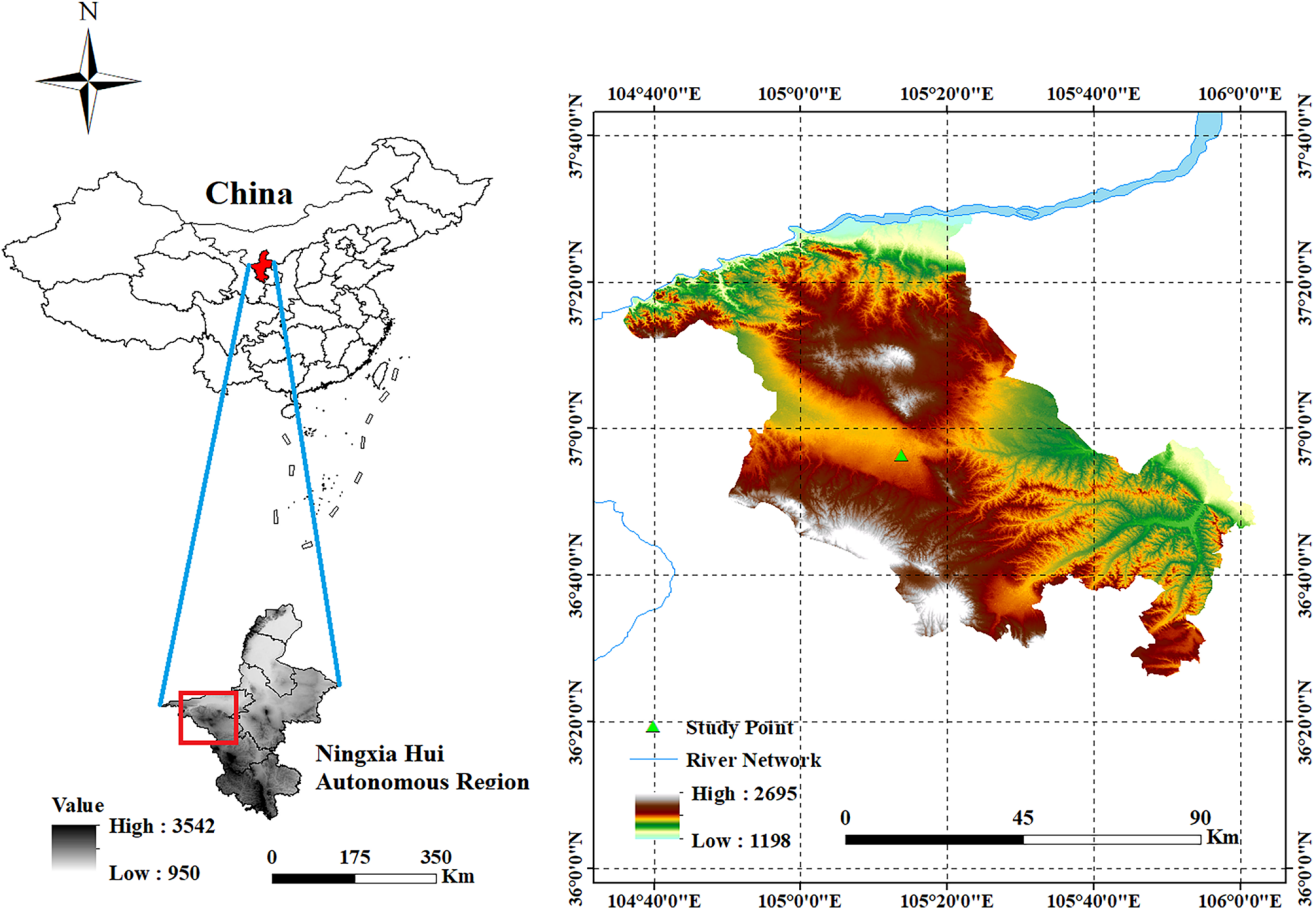
The topographic map of the study area (created using ArcGIS 10.2, https://www.esri.com/en-us/arcgis/about-arcgis/overview).

The study area was the central arid zone of Ningxia, China, which is located at 105° 13′ 44″ E and 36° 56′ 24″ N, with an average altitude of 1740 m^12^. The region has a typical continental monsoon climate with four distinct seasons and variable weather year-round. The winter and spring are dry with wind-blown sand; the summer have large diurnal temperature ranges^13^. The average annual evaporation reaches 2100–2400 mm, which is about ten times the rainfall amount. Ningxia's central dry zone is ecologically vulnerable (see Fig. 1).

The test was performed on grey–green slate with a cryptocrystalline or microscopic scale metamorphic structure^14^, which is primarily composed of quartz, chlorite, calcite, mica, and plagioclase^15^.

### Experiment design

This experiment included an indoor simulation with temperature and particle size as variables. The environmental climate of the test area was consistent with the climate type of the study area. A two-factor four-level proposed level test was used, with sixteen treatments replicated three times for 48 samples (Table 1). The drying temperature was set as factor A, and four levels (44 °C, 36 °C, 28 °C, 20 °C) were set as levels 1, 2, 3, and 4; the drying temperature treatment group test comprised levels A1, A2, A3, and A4. The particle size was set as factor B, and four levels (10–5 mm, 5–2 mm, 2–1 mm, 1–0.5 mm) were set as levels 1, 2, 3, and 4. The particle size treatment group test comprised B1, B2, B3, and B4.

**Table 1:** The combinations of test treatments.

Treatment	1	2	3	4	5	6	7	8
Combination	A1B1	A1B2	A1B3	A1B4	A2B1	A2B2	A2B3	A2B4
Treatment	9	10	11	12	13	14	15	16
Combination	A3B1	A3B2	A3B3	A3B4	A4B1	A4B2	A4B3	A4B4

Coarse soil particles (mainly grey–green slate) make up a large proportion of the soil in the study area due to land degradation. Grey–green slate undergoes a freeze–thaw dry–wet cycle. The mineral particles on the surface or inside break away from the rock to form debris that enters the soil. To clarify the impact of these materials on local soil structure and fertility, this study sets particle sizes of 10–5 mm, 5–2 mm, 2–1 mm, and 1–0.5 mm according to the USDA grading scale, mineral particle size, and the actual conditions of the study area.

The freeze–thaw cycle temperature parameters were determined using ground temperature data from the freeze–thaw period in the study area and freeze–thaw test specifications. The freezing temperature was set to − 20 °C according to the freezing point of rock. The thawing temperature was based on "SL_T264-2020 Code for rock tests in water and hydropower projects": an average value of − 15.4 °C for the cumulative monthly average minimum surface air temperature in winter and spring in the study area (1989–2019). The dissolving temperature was set to 20 °C based on the cumulative monthly average maximum surface air temperature of 18.5 °C in the study area (1989–2019).

The dry–wet cycle temperature parameter was set to reflect the difference in temperatures between irrigation water and the surface temperature during field irrigation in summer and autumn. The average maximum surface temperature in the study area in summer and autumn was 44.1 °C, according to the 30-year annual average maximum surface temperature. The average monthly average surface air temperature over the period was 21.6 °C. The four drying temperature levels were set for the drying temperature control intervals: 44 °C, 36 °C, 28 °C, and 20 °C. Considering local irrigation, the moist temperature is often used as the wet temperature.

The duration of one freeze–thaw and dry–wet cycle (48 h) was based on the "SL_T264-2020 Code for rock tests in water and hydropower projects" and the duration of freeze–thaw and dry–wet periods in the study area over one year. According to the freeze–thaw period (December to May) and dry–wet period (June to November), the freeze–thaw cycle was set to 24 h and the dry–wet cycle was set to 24 h.

According to the specifications of freeze–thaw and dry–wet cycle tests and the physical properties of grey–green slate, a freezing temperature of − 20 ± 2 °C and freezing time of 10 h were used (total water). The dissolution temperature was 20 ± 2 °C, with a time of 14 h. The drying temperature varied: 44 ± 2 °C, 36 ± 2 °C, 28 ± 2 °C, and 20 ± 2 °C. The drying time was 16 h, and soaking was carried out at 4 °C for 8 h. The weathered debris was sampled after 90 cycles to analyze the particle size characteristics.

### Particle size distribution

The particle sizes of the different components of the weathered debris and the distributions of the other particle sizes were determined with the BT-2003 laser particle size distribution instrument produced by Dandong Baite Technology Co. The particle size composition classification adopts the international Udden-Wentworth classification: less than 3.9 μm for clay, 3.9–7.8 μm for very fine silt, 7.8–15.6 μm for fine silt, 15.6–31.2 μm for medium silt, 31.2–62.5 μm for coarse silt, 62.5–125 μm for very fine sand, 125–250 μm is fine sand, and more than 250 μm for medium-coarse sand^16,17^.

The particle size parameter is an absolute value for expressing the particle size characteristics of debris material. Commonly used particle size parameters include the average particle size, standard deviation, kurtosis, skewness, and fractal dimension^18–21^. In this paper, we use the graphical method proposed by Folk and Ward^22^ to calculate the particle size parameters. The specific equations are shown in Table 2.

**Table 2:** The formulas for particle size parameters.

Particle size parameters	Formula
Median particle size (*M*_*z*_)	*Mz* = *Ф*_50_
Average particle size (*M*_*d*_)	*M*_*d*_ = (*Ф*_16_ + *Ф*_50_ + *Ф*_84_)/3
Standard deviation (*σ*)	*σ* = (*Ф*_84_ − *Ф*_16_)/4 + (*Ф*_95_ − *Ф*_5_)/6.6
Skewness (*S*_*K*_)	*S*_*K*_ = (*Ф*_84_ + *Ф*_16_ − 2*Ф*_50_)/2(*Ф*_84_ − *Ф*_16_) + (*Ф*_95_ + *Ф*_5_ − 2*Ф*_50_)/2(*Ф*_95_ − *Ф*_5_)
Kurtosis (*K*_*G*_)	*K*_*G*_ = (*Ф*_95_ − *Ф*_5_)/2.44(*Ф*_75_ − *Ф*_*25*_)

*Φ* = − log 2 *D*, *D* is the particle size of the particle, unit: *mm.*

The fractal dimension of the weathered debris particle size was calculated^23–25^ as follows:

1 $$ \frac{{V_{{(r < R_{i} )}} }}{{V_{T} }} = \left( {\frac{{R_{i} }}{{R_{\max } }}} \right)^{3 - D} $$

where *D* is the fractal dimension of weathered debris size; *r* is the size of weathered debris (μm); *R*_*i*_ is the size of weathered waste of size class *i* (μm); *R*_*max*_ is the extreme value of weathered debris size (μm); *V* (*r* < *R*_*i*_) is the volume fraction of weathered debris with soil size less than *R*_*i*_ (%); *V*_*T*_ is the sum of the volume fraction of each size class (%).

### Elemental content determination

The elemental content was determined using an Agilent 8900 plasma mass spectrometer. Because the solid sample particles are large enough to clog and damage the instrument, they must be dissipated. The microwave digestion extraction system included sample digestion and a DihiBlock ED16 digestion instrument for elemental content measurement after digestion.

## Results and discussion

### Weathered debris particle size composition and particle size parameter change

#### Particle size composition

**Figure 2:**
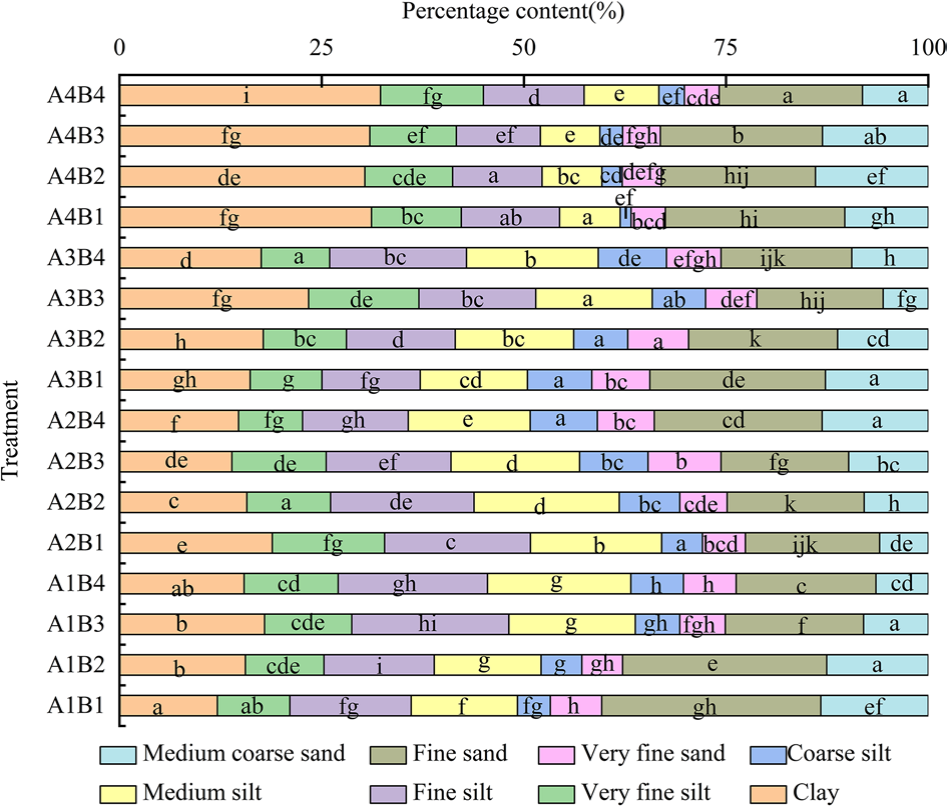
The particle size distribution of weathered debris.

Under different temperatures, the particle size fractions of weathered debris produced by different particle sizes of grey–green slate significantly differed (significance level up to 0.05, Fig. 2). As seen in the figure, the weathered debris of each treatment combination contained relatively more clay, fine silt, and fine sand, and the lowest amount of very fine sand and coarse silt (less than 10%). The clay content of weathered debris in the A4B1, A4B2, A4B3, and A4B4 treatments was higher than that in the remaining treatments, while the silt content in the four treatments was less than that in the remaining treatments. Thus, a smaller particle size of grey–green slate indicates more clay content of weathered debris in the process of freeze–thaw and dry–wet cycles; the possibility of weathering into clay loam is more unusual. On the other hand, grey–green slate can somewhat increase the clay content of the soil, which helps improve the physical and chemical properties of soil and plant growth^26^ alleviate desertification. In all treatments except for A4B1, A4B2, A4B3, and A4B4, the clay, sand, and silt (very fine silt, fine silt, medium silt, and coarse silt) in the weathered debris were all greater than 30%. During the freeze–thaw and dry–wet cycles, slate with smaller particle sizes had a more uniform particle size distribution of weathered debris.

Temperature has a reduced effect on particle size composition variations of grey–green slate debris under freeze–thaw and dry–wet cycles. The particle size relationship between the particle size fraction of grey–green slate and temperature is as follows: when the particle size is 10–5 mm, the content of fine sand and medium sand gradually increases as temperature increase; the content of clay and very fine sand increases and then decreases as temperature increases; and the content of fine sand and very fine sand decreases and then increases with a temperature increase. When the particle size is 5–2 mm, the content of medium silt, coarse silt, and very fine sand first increases and then decreases with a temperature increase. When the particle size is 2–1 mm, the content of fine sand increases gradually as temperature increases, and the content of clay and very fine silt first increases and then decreases with a temperature increase. When the particle size is 1–0.5 mm, the content of clay, very fine silt, and fine silt decreases and then increases with a temperature increase, and the range of very fine sand and medium coarse sand increases and then decreases as temperature increases.

**Figure 3:**
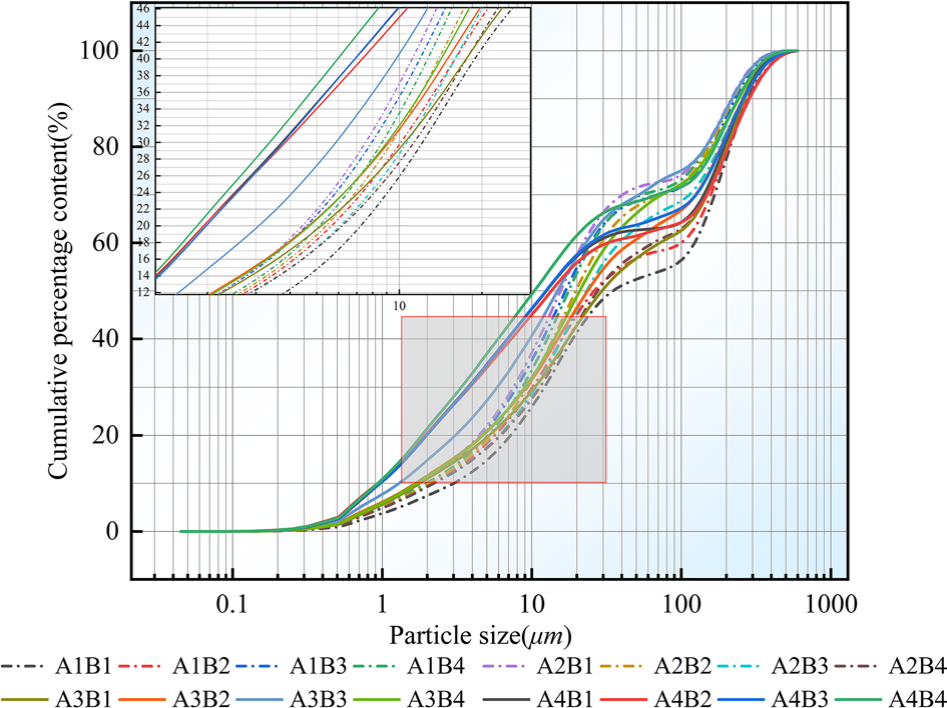
The plot of the cumulative frequency distribution curve.

The cumulative content percentage of the particle size value was used to determine the particle size cumulative frequency distribution curve. The points on the curve represent the probability cumulative percentage sum before the particle size. The range of particle sizes on the curve and the slope of the curve provides an analysis of the size distribution, coarseness, and sorting characteristics of the weathered debris^17^. Figure 3 depicts the frequency distribution curves of the particle size accumulation for various particle sizes of grey–green slate under different temperatures. According to the graph, the frequency distribution curves of the particle size accumulation for each treatment had similar shapes and trends. The slope of the curve was greater for each treatment in the 0.5 μm to 30.75 μm range, with the cumulative slope of the curve under treatments A4B2, A4B3, and A4B4 being approximately 1, indicating that these three treatments had better sortability in the 0.5 μm to 30.75 μm particle size range. The curve is flatter for 30.7–102.4 μm and the slope is almost zero, indicating poor sortability across treatments in this particle size range. The curve shows a steep increase for 102.4–413 μm, indicating a higher sand content and better sorting under each treatment.

#### Variation of particle size parameters

**Figure 4:**
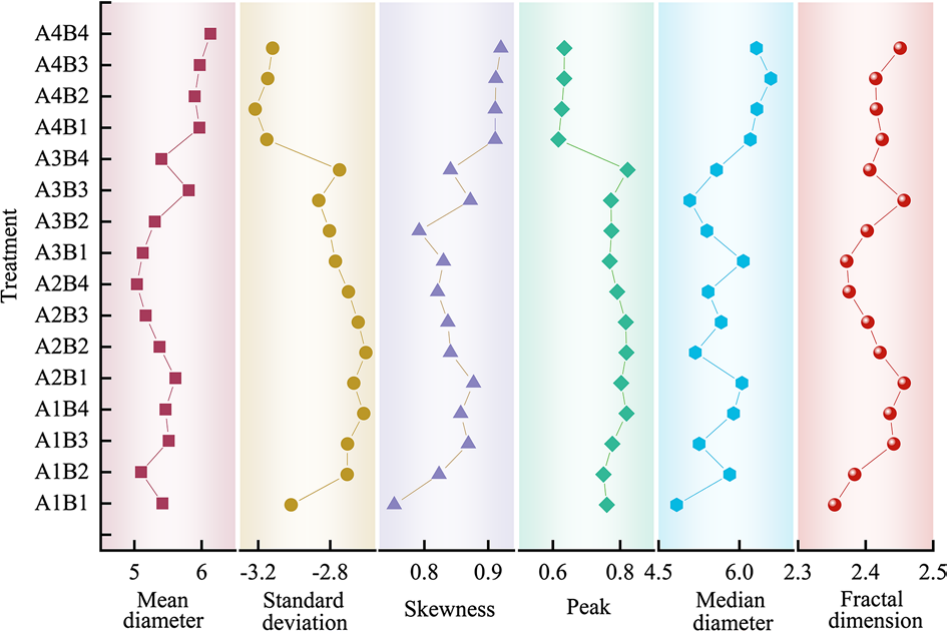
The variation of particle size parameters of weathered debris.

According to Table 2 and Eq. (1), the particle size parameter calculation formula was derived from the particle size parameters of weathered grey–green slate debris under different temperatures and particle size treatments (see Fig. 4). As seen from the figure, the average particle size of the weathered debris of grey–green slate under each treatment was 5.52 *Ф* (silt grain class 4 *Ф*–8 *Ф*). At 44 °C and 36 °C, the average particle size of weathered debris increased, decreased, and then increased as the particle size of the grey–green slate became smaller. At 28 °C and 20 °C, the average particle size of weathered debris increased, then decreased with a decrease in the particle size of grey–green slate. The average particle size was largest in the A4B4 treatment and decreased as follows: A4B4 > A4B3 > A4B1 > A4B2 > A3B3 > A2B1 > A1B3 > A1B4 > A1B1 > A3B4 > A2B2 > A3B2 > A2B3 > A3B1 > A1B2 > A2B4.

The median particle size distribution of the weathered debris in each treatment ranged from 4 *Ф* to 7 *Ф*, with the silt grade of 4 *Ф–*8 *Ф*. Under any temperature treatment, the median particle size of weathered debris with the particle size of grey–green slate was consistent with the average particle size.

Weathering debris skewness varied from 0.7 to 0.95. Overall, the debris had highly positive skewness without symmetry, with an average value of 0.85. The maximum skewness value occurred in the A4B4 treatment, and the minimum skewness value occurred in the A1B1 treatment. Overall, the size of weathered debris in each treatment was concentrated in the coarse range. Weathered debris had a standard deviation of − 3.5 to − 2.5, with a fluctuation of 1 and a mean value of − 2.84. The kurtosis variation of weathered debris ranged from 0.65 to 0.85, with a mean value of 0.75. The low kurtosis values of the curves for the 16 treatments indicate that the particle size frequency distribution curves are mostly broad- or multi-peaked. The kurtosis of the weathering debris frequency curves of 1–0.5 mm and 5–2 mm grain sizes gradually decreased with increasing temperature. 44 °C, 36 °C, and 28 °C showed a trend of increasing and then decreasing weathering debris kurtosis with a decrease in the grey–green slate grain size.

In general, the fractal dimension can categorize the distribution and homogeneity of soil particle size as well as reflect soil fertility. A greater fractal dimension corresponds to more fine particles in the soil^27,28^. The mean value of the weathered debris fractal dimension was 2.41, which is distributed between 2.35 and 2.46. The most significant values of the weathered debris fractal dimension occurred in A2B1 and A3B3, indicating that the two treatments had a higher content of fine particles. The cumulative content of clay, very fine silt, fine silt, medium silt, and coarse silt in the two treatments was greater than 72%, which was higher than that in the remaining treatments.

### Relationship between fractal dimension and particle size composition of weathered debris

**Figure 5:**
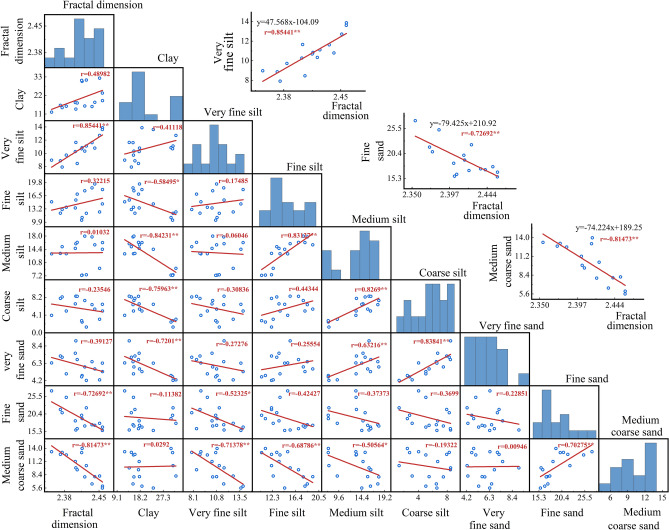
The correlation between fractal dimension and particle size composition. (*indicates a significant difference at the *P* < 0.05 level, ** indicates a highly significant difference at the *P* < 0.01 level).

The fractal dimension of weathered debris for different treatments was regressed against the particle size composition (Fig. 5).

The figure shows that the fractal dimension of weathered debris is linearly related to the particle size composition in the first order. The fractal dimension was significantly positively correlated (*P* < 0.01) with the content of very fine sand (correlation coefficient of 0.85441) and highly negatively correlated (*P* < 0.01) with the content of fine and medium coarse sand (correlation coefficients of − 0.72692 and − 0.81473, respectively). The fractal dimension was positively correlated with the content of clay, fine silt, and medium silt, and it was negatively correlated with the content of coarse silt and very fine sand. The correlation between the fractal dimension of weathered debris and the content of clay, very fine silt, fine sand, and medium-coarse sand is roughly the same as that found by other scholars^29^ studying the correlation between soil fractal dimension and particle size composition. Specifically, the fractal dimension had a positive correlation with clay and very fine sand and a negative correlation with fine sand and medium-coarse sand. There is a correlation between the fractal dimension and each particle size composition of weathered debris, which shows that the fractal dimension can characterize the soil's particle size composition. The diagonal histogram in Fig. 5 showed the distribution of fractal dimension and particle size composition under each treatment, which shows that the distribution of fractal dimension and particle size composition under each treatment is more discrete and less concentrated. The fine silt has a highly significant positive correlation with the medium silt, the medium silt has a highly significant positive correlation with the very fine sand and the coarse silt, and the coarse silt has a highly significant positive correlation with the very fine sand, which shows that the fine silt, the medium silt, the very fine sand and the coarse silt in the particle size composition of the weathered debris have a relationship that promotes each other's production. There was a highly significant negative correlation between very fine silt and medium coarse sands, and a highly significant negative correlation between fractal dimension and medium coarse sand content, as the larger the fractal dimension, the more fine particles in the fragments.

### Relationship between fractal dimension and mineral elements

The weathering debris formed during the freeze–thaw dry–wet cycle of grey–green slate is partly due to the formation of rock surface fragmentation and leaching, i.e., the element content affects the particle size composition of the weathering debris to an extent. Partial least squares regression (PLSR) was used to screen the mineral element factors affecting the fractal dimension of weathered debris to clarify the influence of each mineral element on the particle size composition of weathered debris.

PLSR is a new multivariate regression analysis method that combines the benefits of multiple linear regression analysis, typical correlation analysis, and principal component analysis. The method can represent the explanation of the dependent variable by the independent variable^30^. The projection importance (VIP) of each mineral element content on the fractal dimension of weathered debris was calculated using B, Mn, Fe, Ni, Cu, Zn, Ca, Mg, P, K, S, Si, Na, Mo, N, and Cl mineral element content as independent variables and weathered debris fractal dimension as dependent variables. The stepwise screening of the respective variables was carried out based on the size of the VIP value of the full PLSR model. A VIP value greater than 0.8 usually indicates that the independent variable has a greater influence on the dependent variable. A VIP value greater than 1 indicates that the independent variable has a large influence on the dependent variable. The mineral element factors influencing the fractal dimension of weathered debris are screened based on VIP values greater than 1.

**Figure 6:**
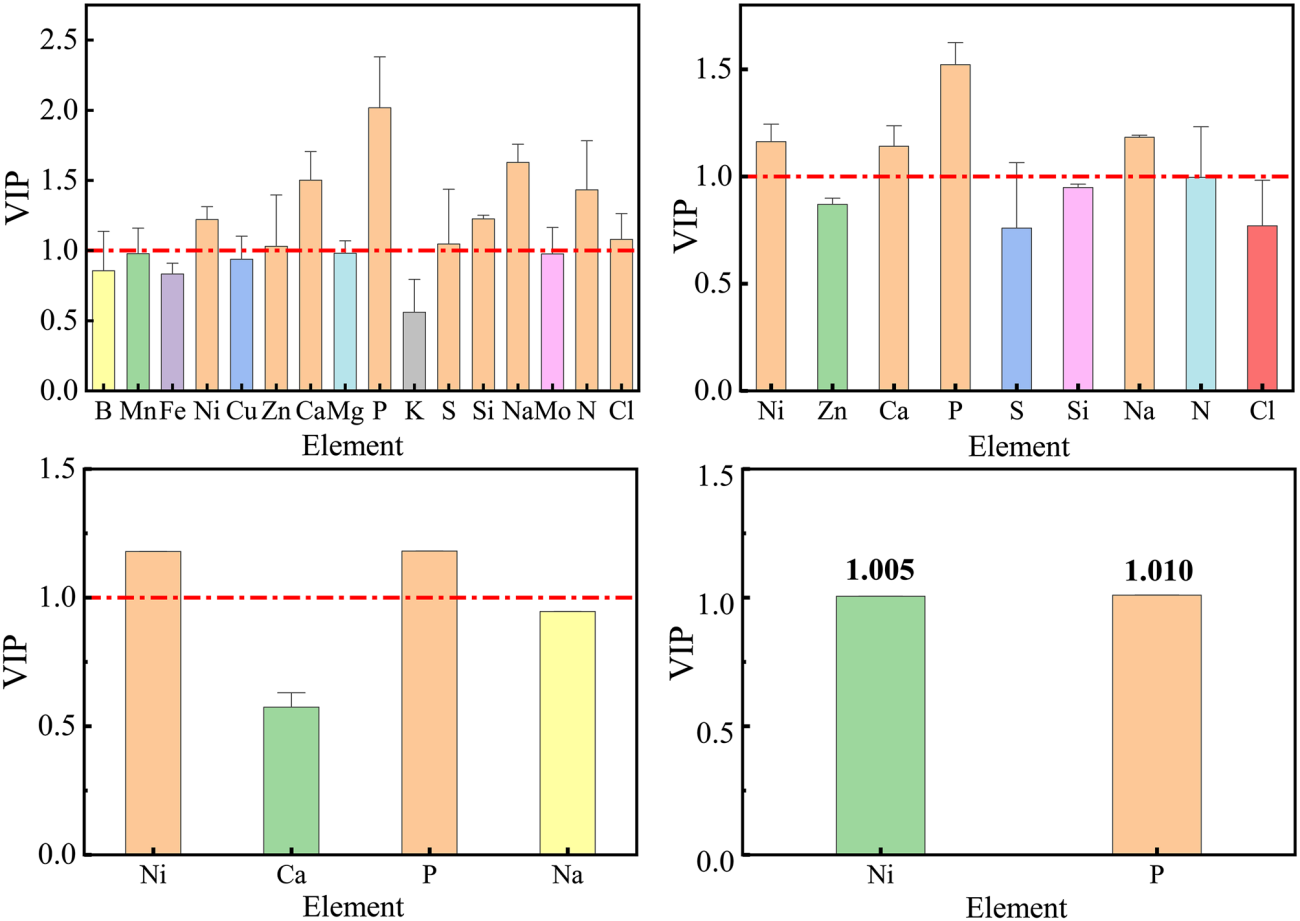
The projected importance (VIP) values of mineral elements.

Figure 6 depicts the stepwise screening of the mineral element factor. The screening was terminated when the VIP values of all independent variables were greater than 1. There were four screenings in total. The VIP values of Ni, Zn, Ca, P, S, Si, Na, N, and Cl in the first screening step were more significant than 1, which indicates that these elements among the 16 mineral elements had a greater influence on the fractal dimension of weathered debris. Mineral elements with VIP values more significant than 1 were screened again, and the results indicate that the VIP values of Ni, Ca, P and Na were more critical than 1. The third screening step showed that the VIP values of Ni and P were higher (1.180 and 1.181, respectively). After screening, the Ni and P VIP values were more significant than 1. At this time, the screening was stopped, and the primary mineral elements affecting the fractal dimension of weathered debris were extracted (i.e., P and Ni).

Combining the VIP values of each mineral element, the magnitudes of the influence of 16 mineral elements on the fractal dimension of weathered debris were largest to smallest as follows: P, Ni, Na, Ca, N, Si, Zn, Cl, S, Mg, Mn, Mo, Cu, B, Fe, and K.

### Analysis of potential environmental factors affecting the fractal dimension of weathered debris

**Figure 7:**
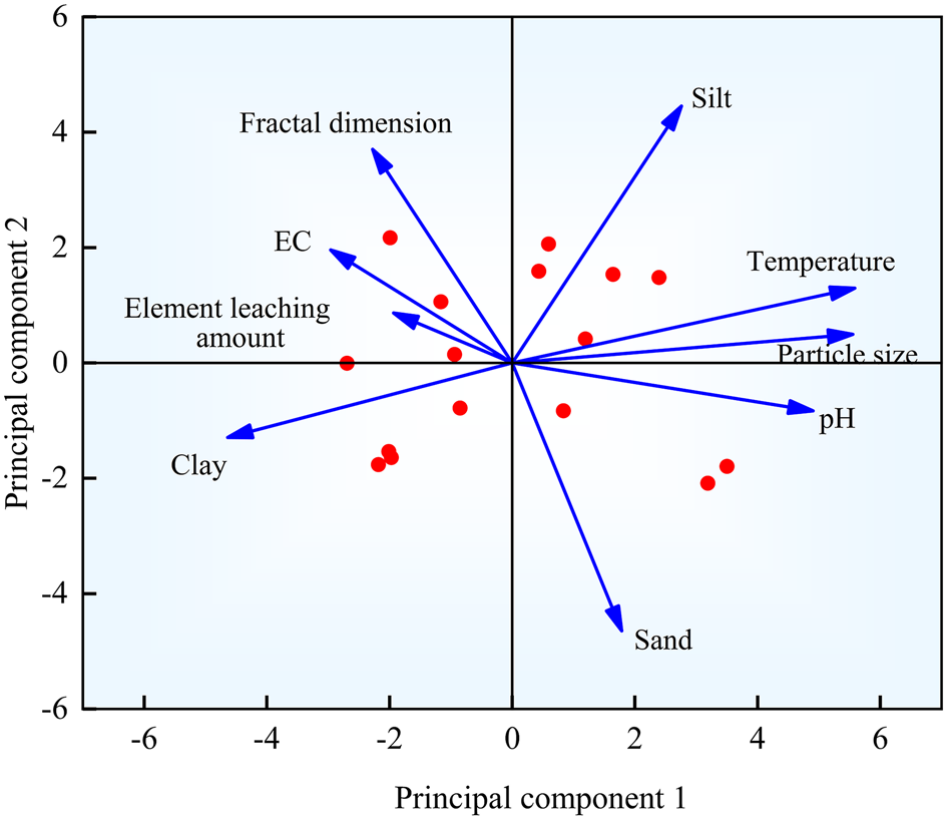
The principal component analysis diagram.

Principal component analysis (PCA) analyzed the potential environmental factors affecting the fractal dimension of weathered debris (see Fig. 7). The variance contribution of the first central component (PC1) was 44.7%, and the variance contribution of the second principal component (PC2) was 25.5%, resulting in a representative cumulative variance contribution of 70.2%. The fractal dimension vector of weathered debris forms an acute angle with the electrical conductivity (EC) and elemental leaching vectors, as shown in Fig. 5. The direction is consistent, indicating a positive relationship between fractal dimension, EC, and elemental leaching. The relationship between the fractal dimension and EC is more robust than the relationship between elemental leaching. The fractal dimension correlated negatively and weakly with particle size, temperature, and pH. Weathered debris formation is generally influenced by EC, elemental leaching, temperature, grain size, and pH.

## Discussion

### Particle size distribution characteristics of weathered debris

Analyzing the particle size composition characteristics of weathered debris from 16 Grey-green slates leads to the conclusion that the content of very fine sand and silt in weathered debris is relatively low under each treatment, and the difference between particle size classes is more significant under different treatments. The main reason for this difference is that the higher the temperature, the greater the stress generated by the expansion and contraction of the rock, and the greater the possibility of rock fragmentation^31^. However, this result may also be due to temperature differences in the water content of the environment in which the rock is located^32^ or the degree of weathering and fragmentation of the rock. Thus, each treatment of weathering debris particle size content is different. To clarify the similarity between the particle size composition of the weathered debris under the freeze–thaw dry–wet cycle and that of the actual field soil, particle size measurements were carried out at the test site of Lycium barbarum (G) after sampling (Table 3).

**Table 3:** The particle size composition of field soil.

Sample	Clay	Very fine silt	Fine silt	Medium silt	Coarse silt	Very fine sand	Fine sand	Medium-coarse sand
G1	9.27	3.99	5.72	7.86	12.37	26.02	29.28	5.49
G2	12.79	6.36	7.45	8.72	9.99	24.53	25.17	4.99
G3	16.25	6.42	9.14	10.18	9.85	20.79	21.60	5.77
G4	7.65	3.29	4.70	7.73	12.84	31.74	27.07	4.98
G5	10.43	4.08	5.52	9.72	12.28	25.95	26.09	5.93
G6	11.82	5.01	7.06	9.31	13.69	22.14	26.04	4.93
Average	11.37	4.86	6.60	8.92	11.84	25.20	25.88	5.35

Table 3 shows that the soil particle size of lycium barbarum is primarily very fine sand and fine sand, with these two particle sizes accounting for 50% of the total, followed by coarse soil particles. Comparing the particle size compositions of weathered debris in each indoor test, very fine sand and fine sand were most abundant for A1B1 and A1B2: these two treatments had very fine sand and fine sand contents greater than 30%; the remaining treatments had less than 30%. The contents of weathered debris clay, very fine silt, and fine silt under each treatment were higher than those of LBP, which shows that the weathered debris contains more fine particles than LBP. The particle size composition of the weathered waste is more delicate than the actual soil particle size, indicating that the grey–green slate has some influence on the soil particle size composition during the freeze–thaw weathering in winter/spring and dry and wet cycles in summer/autumn. Measures to accelerate the weathering of the grey–green slate can help improve the soil structure if the soil fertility can be modified.

By calculating the particle size parameters of weathered debris under each treatment, it is clear that the average particle size of weathered waste (1–0.5 mm) under each temperature treatment was larger than other particle sizes. The larger the average particle size, the larger the average kinetic energy of the transport medium^33^. Therefore, the average crushing kinetic energy of 1–0.5 mm particle sizes during the freeze–thaw and dry–wet cycles was greatest among those studies. The standard deviation of weathered debris varied between − 3.5 and − 2.5. The sortability of weathered debris under each treatment was excellent according to the table of particle size sortability by FOLK^22^. Thus, the source of weathered debris was relatively homogeneous because only water and grey–green slate are involved in weathering throughout the freeze–thaw and dry–wet cycles. The distribution of fractal dimensions of each type of weathered debris in the experiment was approximately the same as that of the soil particle size fractal dimension in the desertification area of Ningxia^34,35^. The actual field soil fractal dimension distribution range was 2.08–2.25. The content of fine particles in the field soil was comparatively lower, which shows that the weathering fragmentation of coarse-grained grey–green slate on the surface in the dry farmland of the central Ningxia arid zone can increase the content of fine particles and modify the soil’s water retention capacity^36^.

### Fractal dimension of weathered debris

The fractal dimension of weathered debris in grey–green slate has a highly significant positive correlation with the content of very fine sands and a highly significant negative correlation with the content of fine sands and mediμm-coarse sands. More fine particles in weathered debris result in a larger fractal dimension. More coarse particles result in a smaller fractal dimension. The fractal dimension can describe the distribution of the material in the weathered debris.

Weathered debris is formed during freeze–thaw and dry–wet cycles by contracting and expansion forces, as well as other factors; it is typically composed of many types of minerals^37^. The debris particle size is influenced by the elemental content. The smaller the particle size composition in weathered debris, the larger the fractal dimension. The rougher the texture, the higher the content of mineral elements such as P, N, and K, which catalyze soil fertility. P and Ni were found to have the greatest influence on the fractal dimension of weathered debris in this study. The regression equation of fractal dimension and these two elements was calculated as D = 0.019P-0.01Ni using least squares regression. In other words, we estimated the fractal dimension of weathered debris and then determined the particle size distribution.

A larger EC of the environment in which the grey–green slate is located correlates with a larger fractal dimension of weathered debris. A larger EC value corresponds to a higher salt content, a weaker cementation between the particles in the freeze–thaw and dry-wet cycles of grey–green slate, a relatively more significant degree of fragmentation, and coarser debris particles. The fractal dimension of weathered debris was found to be negatively correlated with pH, which is consistent with the relationship between fractal dimension and pH of the soil in the wind-sand area of Yanchi County, Ningxia^38^. Specifically, finer textured particles result in a more alkaline environment.

## Conclusion

1. After the freeze–thaw and dry-wet cycles, the size content of the weathered debris under each treatment was in the order of clay, fine sand, fine silt, medium silt, very fine silt, medium coarse sand, coarse silt, and very fine sand, which to some extent contributed to the improvement of the soil particle composition and increased the content of the fine particle fraction in the soil.
2. Grain size was the main influence on the particle size composition of weathered grey–green slate, with the temperature being a secondary factor, and the particle size distribution curve was extremely positively skewed with poor symmetry and non-uniform distribution.
3. The fractal dimension of weathered debris has a highly significant positive correlation with the content of very fine silt and a highly significant negative correlation with the content of fine sand and medium coarse sand.
4. The nature (elemental content, conductivity, pH) and grain size of the grey–green slate are influential factors in the fractal dimension of the weathered debris.

## Data Availability

The datasets used and/or analysed during the current study available from the corresponding author on reasonable request.
